# Cardiac glycosides display selective efficacy for STK11 mutant lung cancer

**DOI:** 10.1038/srep29721

**Published:** 2016-07-19

**Authors:** Nayoung Kim, Hwa Young Yim, Ningning He, Cheol-Jung Lee, Ju Hyun Kim, Jin-Sung Choi, Hye Suk Lee, Somin Kim, Euna Jeong, Mee Song, Sang-Min Jeon, Woo-Young Kim, Gordon B. Mills, Yong-Yeon Cho, Sukjoon Yoon

**Affiliations:** 1 Center for Advanced Bioinformatics & Systems Medicine, Sookmyung Women’s University, Seoul, Republic of Korea; 2 Department of Biological Sciences, Sookmyung Women’s University, Seoul, Republic of Korea; 3 College of Pharmacy, The Catholic University of Korea, Gyeonggi-do, Republic of Korea; 4 College of Pharmacy, Ajou University, Gyeonggi-do, Republic of Korea; 5 RCCFC, College of Pharmacy, Sookmyung Women’s University, Seoul, Republic of Korea; 6 Systems Biology, University of Texas, MD Anderson Cancer Center, Houston, Texas, USA

## Abstract

Although *STK11* (LKB1) mutation is a major mediator of lung cancer progression, targeted therapy has not been implemented due to *STK11* mutations being loss-of-function. Here, we report that targeting the Na^+^/K^+^-ATPase (ATP1A1) is synthetic lethal with *STK11* mutations in lung cancer. The cardiac glycosides (CGs) digoxin, digitoxin and ouabain, which directly inhibit ATP1A1 function, exhibited selective anticancer effects on *STK11* mutant lung cancer cell lines. Restoring *STK11* function reduced the efficacy of CGs. Clinically relevant doses of digoxin decreased the growth of *STK11* mutant xenografts compared to wild type *STK11* xenografts. Increased cellular stress was associated with the *STK11*-specific efficacy of CGs. Inhibiting ROS production attenuated the efficacy of CGs, and STK11-AMPK signaling was important in overcoming the stress induced by CGs. Taken together, these results show that *STK11* mutation is a novel biomarker for responsiveness to CGs. Inhibition of ATP1A1 using CGs warrants exploration as a targeted therapy for *STK11* mutant lung cancer.

The tumor suppressor *STK11* is inactivated in 30% of lung cancer cases (∼20% of lung adenocarcinoma (LUAD) cases)^1^. Despite the importance of STK11 in lung cancer^1^,^2^,^3^ and the observation that therapeutic approaches for other types of cancer harboring genetic alterations have impacted patient care (Supplementary Table S1), targeted therapeutic approaches have not yet been implemented for lung cancer harboring *STK11* mutations, a loss-of-function aberration. It remains challenging to identify effective targets with a clear mechanistic explanation for the concerted role of a target and a loss-of-function mutation.

NCI60 screening datasets include a set of GI50 data consisting of ~50,000 chemicals screened in 59 diverse cancer cell lines^4^,^5^. This dataset provides a unique opportunity to identify the association of chemical responses with lineages and mutations of cancer cells. Because several cell lines in the NCI60 panel harbored a loss-of-function mutation in *STK11*, we were able to investigate the specific and sensitive responses of *STK11* mutant cell lines to chemicals in comparison with other cell lines. Interestingly, *STK11* mutant lung cell lines were found to exhibit specific sensitivity to a group of cardiac glycosides (CGs).

CGs are a family of compounds (including digoxin^6^, digitoxin^7^, and ouabain^8^) that have been widely used for the treatment of congestive heart failure and arrhythmia^9^. CGs regulate the intracellular sodium and potassium ion concentrations by inhibiting the enzyme Na^+^/K^+^-ATPase (ATP1A1)^10^,^11^. Anticancer effects of CGs have been reported, but these compounds have not entered patient care in part due to a lack of selective markers of their bioactivity^12^.

In the present study, we demonstrated the association of CG efficacy with the mutational status of *STK11* by collectively analyzing NCI60 chemical screen data and Achilles^13^ loss-of-function screen data. *In vitro* validation confirmed that restoring *STK11* function decreased the cellular response to CGs in a wide variety of *STK11* mutant lung cancer cell lines. We further investigated the *in vivo* efficacy of CGs using mouse xenograft models and demonstrated that clinically relevant doses of CGs exerted selective effects on *STK11* mutant tumors. Proteomic analysis using reverse phase protein arrays (RPPAs) and molecular mechanism studies provided insights into the synthetic lethality of CGs (with or without chemotherapeutic agents) with *STK11* mutant cells.

## Results and Discussion

### Association of the effect of cardiac glycosides with *STK11* mutation

**Figure 1:**
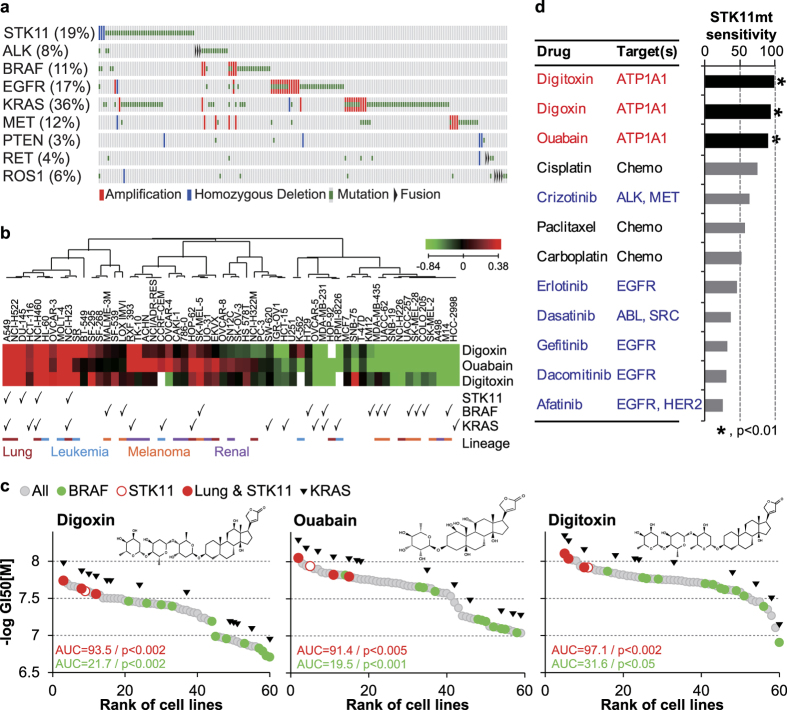
*STK11* mutation-specific responses (GI_50_ profiles) of CGs. (**a)** Major genetic alterations in LUAD as summarized by TCGA using cBioPortal. (**b)** The lineage and mutation specificities of the responses of NCI60 cell lines to CGs. Median fold-changes in the GI_50_ values were used to generate the heatmap profile. (**c)** *STK11*-specific responses to the three tested CGs. For each drug, the rank order of the −log(GI_50_) values for 59 NCI60 cell lines was used to calculate the area under the curve (AUC) and p-values for the specified lineage or mutation groups (indicated with colors). (**d)** Comparison of CGs with LUAD drugs for selectivity to *STK11* mutant lung cancer cells. To compare *STK11*-specific sensitivity among the tested drugs, the AUC of −log(GI_50_) for each drug was calculated for the *STK11* mutant cell lines relative to all NCI60 cell lines. Detailed mutation information of STK11 in cell lines are found in Supplementary Table S2.

The characterization of genetic alterations in LUAD has enabled the discovery of new molecular targets and therapeutic applications^14^,^15^,^16^. The presence of mutations or rearrangements in genes such as *ALK*, *BRAF*, *EGFR*, *KRAS*, *MET*, *PTEN*, *RET*, and *ROS1* predicts sensitivity and clinical treatment outcome for agents targeting aberrations in LUAD^17^. Targeted agents against oncogenic mutations such as *ALK*, *EGFR*, and *KRAS* have been widely reported as successful therapeutic approaches in non-small cell lung cancer^18^,^19^. *STK11* loss-of-function mutation is mutually exclusive from these oncogenic mutations in The Cancer Genome Atlas (TCGA)-derived LUAD samples, except for a subset of patient samples harboring concurrent mutations in *KRAS* and *STK11* (Fig. 1a). The mutation rate of *STK11* increases from the early to late stages of LUAD (Supplementary Fig. S1), consistent with the observation that STK11 is a modulator of lung cancer metastasis^2^.

To investigate the association of *STK11* mutations with cellular responses to a wide variety of drugs, we used publically available high-throughput drug screening data from the NCI60 cancer cell line dataset^5^,^20^. Strikingly, digoxin, digitoxin, and ouabain demonstrated selective activity on *STK11* mutant lung cancer cell lines based on the response profile of these drugs across NCI-60 cell lines (Fig. 1b,c). In contrast, *BRAF* mutations were associated with marked resistance to CG treatment. Although *KRAS* mutations in the absence of *STK11* mutations were weakly associated with resistance to CG treatment, *KRAS* mutations did not alter the sensitivity of *STK11* mutant cell lines to CGs. This result is important because concurrent *STK11* and *KRAS* mutations are common in LUAD tissue samples (Fig. 1a). In addition, we performed cell line enrichment analysis^21^ to compare the sensitivity and selectivity of CGs with 9 clinically tested therapeutic agents for LUAD using *STK11* mutant lung cancer cell lines (Fig. 1d). Notably, only the response to CGs was significantly associated with *STK11* mutations. Furthermore, *STK11* mutant cell lines were resistant to other targeted drugs used for LUAD, and the efficacy of chemotherapeutic agents demonstrated no correlation with *STK11* mutational status. This result is consistent with the selectivity of CG-mediated inhibition of ATP1A1 to *STK11* mutant cell lines.

### *STK11*-dependent anticancer effect of CGs

**Figure 2:**
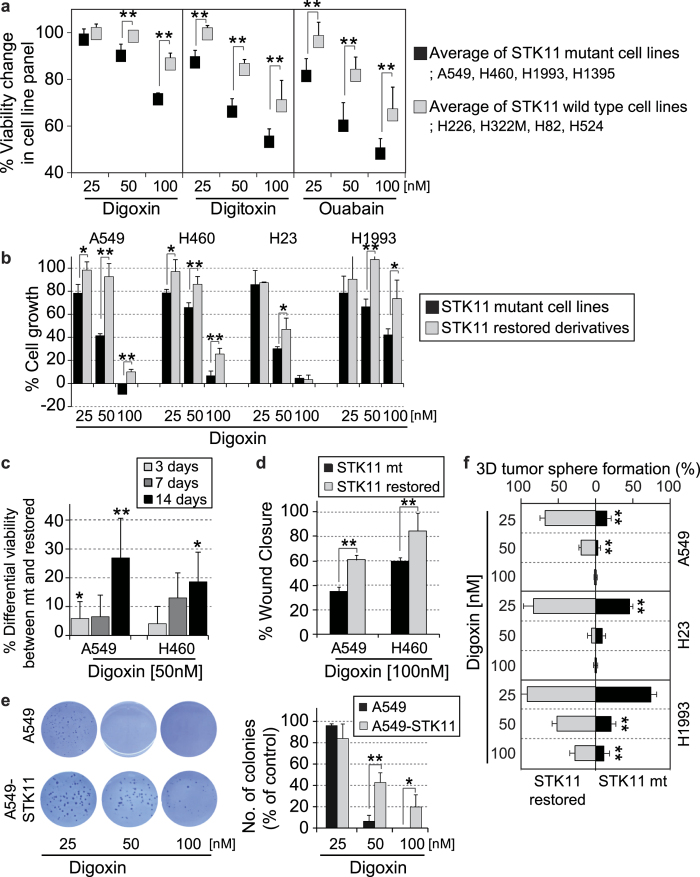
*In vitro* validation of the efficacy of CGs on *STK11* mutant cancer cells. (**a)** Comparison of the change in cell viability induced by CG treatment between *STK11* mutant and wild type cell lines. A total of 8 different lung cell lines were treated with a CG at 25, 50, or 100 nM for 3 days. (**b)** Comparison of digoxin sensitivity between *STK11* mutant lung cell lines and their *STK11*-restored derivatives. Each cell line was treated with 25, 50, or 100 nM digoxin for 3 days. The percentage growth was calculated based on time zero and control growth data (See details in Methods section). Percentage growth of 0 means no net growth and −100 was indicated when all cells are killed. (**c)** Long-term effect of digoxin treatment on the cell viability of *STK11* mutant cell lines (A549 and H460) and their *STK11*-restored derivatives (A549-STK11 and H460-STK11). The viability of each *STK11* mutant cell line relative to its respective *STK11*-restored derivative was monitored following treatment with 50 nM digoxin for 3, 7 or 14 days. (**d)** Changes in cell migration after 100 nM digoxin treatment (24 hours) in the *STK11* mutant (A549 and H460) and *STK11*-restored (A549-STK11 and H460-STK11) cell lines. (**e)** Effect of digoxin treatment on colony formation in the A549 and *STK11*-restored A549 cell lines. Cells were treated with 25, 50, or 100 nM digoxin for 2 weeks, followed by staining with 0.05% crystal violet and colony counting. (**f)** Changes in tumor sphere formation after digoxin treatment in the *STK11* mutant (A549, NCI-H23, and NCI-H1993) and their *STK11*-restored derivative cell lines. Cells were treated for 5, 5, and 4 days, respectively, with 25, 50, or 100 nM digoxin. The ectopic expression of LKB1 protein in each *STK11*-restored cell line was validated (Supplementary Fig. S2). The rates of cell viability, wound closure and colony formation were calculated using DMSO as a control for each inhibitor. *p < 0.05 and **p < 0.01 (Student’s t-test) between the compared groups.

Confirming the NCI60 screening results, we demonstrated that digoxin, digitoxin, and ouabain exerted a significant dose-dependent selective inhibition of the viability of *STK11* mutant cells compared to *STK11* wild type cells in a collection of lung cancer cell lines (Fig. 2a). To determine the selectivity of CGs to cells harboring mutant *STK11*, we introduced wild type *STK11* into A549, H23, H1993 LUAD and H460 large-cell lung carcinoma (LCLC) cells (lacking wild type *STK11*). Introducing wild type *STK11* into these cells increased cell viability following treatment with each CG (Fig. 2b and Supplementary Fig. S3a,b). This result indicated that *STK11* function was directly linked to the sensitivity of cancer cells to CGs. In addition, the effects of CGs on *STK11* mutant cells increased in a time-dependent manner (Fig. 2c and Supplementary Fig. S3c,d). CGs not only decreased cell viability but also decreased BrdU incorporation and wound closure in *STK11* mutant cells; these results indicated the effects of CGs on both cell proliferation and migration (Fig. 2d and Supplementary Fig. S3e,f). Their effects on cell proliferation and migration were coordinately decreased by the introduction of wild type *STK11*. Furthermore, we performed 3D *in vitro* assays to mimic the *in vivo* tumor environment. Treatment with digoxin selectively inhibited anchorage-independent growth of *STK11* mutant cells compared to *STK11*-restored cells, regardless of the presence (Fig. 2e) or absence (Fig. 2f) of serum. In summary, the ability of exogenous wild type *STK11* to reverse the effects of CGs suggests that the activity of CGs depends on *STK11* mutations, but not *KRAS* mutations, which frequently occur concurrently with *STK11* mutations (Fig. 1a).

### *STK11*-dependent anticancer effect of *ATP1A1* gene knockdown

**Figure 3:**
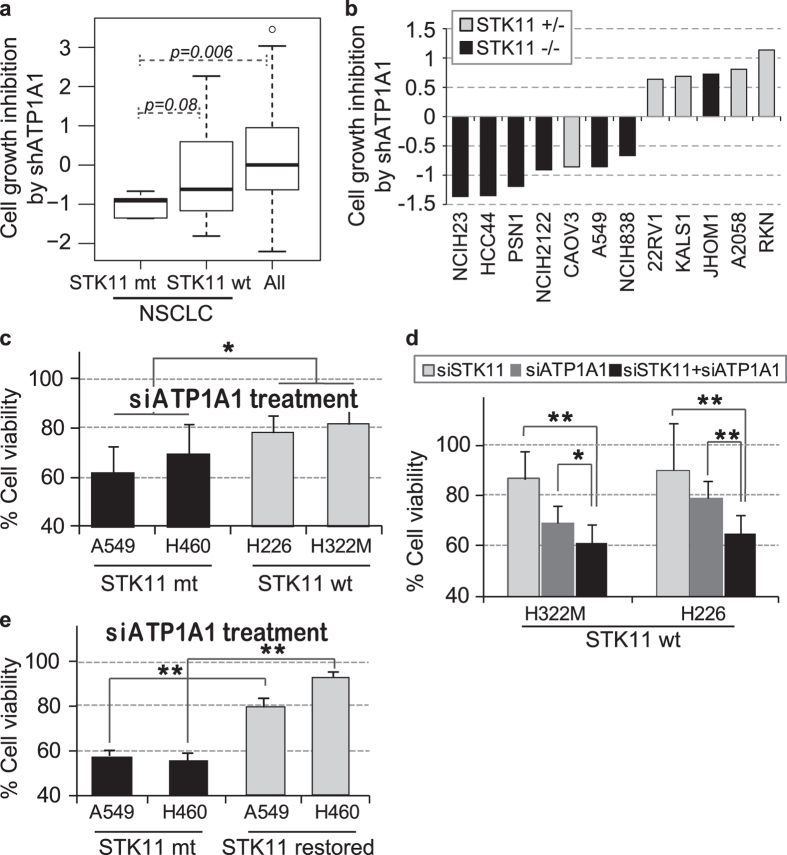
*STK11* mutation-specific effect of *ATP1A1* gene knockdown on cell viability. (**a)** Effect of shATP1A1 transfection on 216 distinct cancer cell lines (raw data retrieved from Project Achilles). The effect of *ATP1A1* knockdown was compared among 3 categories of cell lines: *STK11* mutant NSCLCs (NCI-H23, HCC44, NCI-H2122, A549, and NCI-H838), *STK11* wild type NSCLCs, and all 216 cell lines. (**b)** Comparison of the effect of shATP1A1 between *STK11* homozygous mutant and *STK11* heterozygous mutant cell lines (raw data retrieved from Project Achilles). (**c)** *In vitro* validation of the change in cell viability induced by siATP1A1 treatment (72 hours) in *STK11* mutant and wild type lung cell lines. (**d)** Effect of *ATP1A1* and *STK11* double knockdown (72 hours) on the viability of *STK11* wild type (NCI-H322M and NCI-H226) cells. (**e)** Effects of siATP1A1 (72 hours) on lung cell lines harboring an *STK11* mutation (A549 and H460) compared to *STK11*-restored (A549-STK11 and H460-STK11) derivatives. The knockdown efficacy of *ATP1A1* and *STK11* siRNA in each cell line was evaluated (Supplementary Fig. S4). The rate of cell viability was calculated using siNC as a control for each target siRNA. *p < 0.05 and **p < 0.01 (Student’s t-test) between the compared groups.

All of the tested CGs, digoxin, digitoxin and ouabain, exhibit highly selective, nano-molar-scale activity on ATP1A1^22^. To examine whether the selectivity of CGs to *STK11* mutant cells is dependent on ATP1A1 activity, we first checked publically available high-throughput shRNA screening data for 216 cancer cell lines; this dataset was derived from Project Achilles^13^. shATP1A1 significantly inhibited the growth of five different *STK11* mutant NSCLC cell lines compared to the *STK11* wild type cell lines (Fig. 3a). Furthermore, loss of heterozygosity in *STK11* mutation markedly increased the inhibitory effect of shATP1A1 on a wide range of cancer cell lines, and this result indicated the dependency of *ATP1A1* inhibition on the loss of *STK11* function (Fig. 3b). For experimental validation, we silenced *ATP1A1* expression in both *STK11* mutant (A549 or H460) and *STK11* wild type (H226 or H322M) cell lines. *ATP1A1* knockdown significantly decreased cell viability in the *STK11* mutant cell lines but not in the *STK11* wild type cell lines (Fig. 3c), suggesting that CG activity is dependent on *ATP1A1* inhibition. In addition, silencing both *STK11* and *ATP1A1* expression in H226 and H322M cells, which harbor wild type *STK11*, decreased cell viability to a greater extent than either *STK11* or *ATP1A1* knockdown alone (Fig. 3d). Moreover, introducing wild type *STK11* decreased the effect of *ATP1A1* knockdown on the A549 and H460 cell lines, which harbor *KRAS* mutation, further supporting the dependence of *STK11* mutant cells on ATP1A1 activity (Fig. 3e).

### Effect of combination of CGs with other therapeutic agents on *STK11* mutant cells

**Figure 4:**
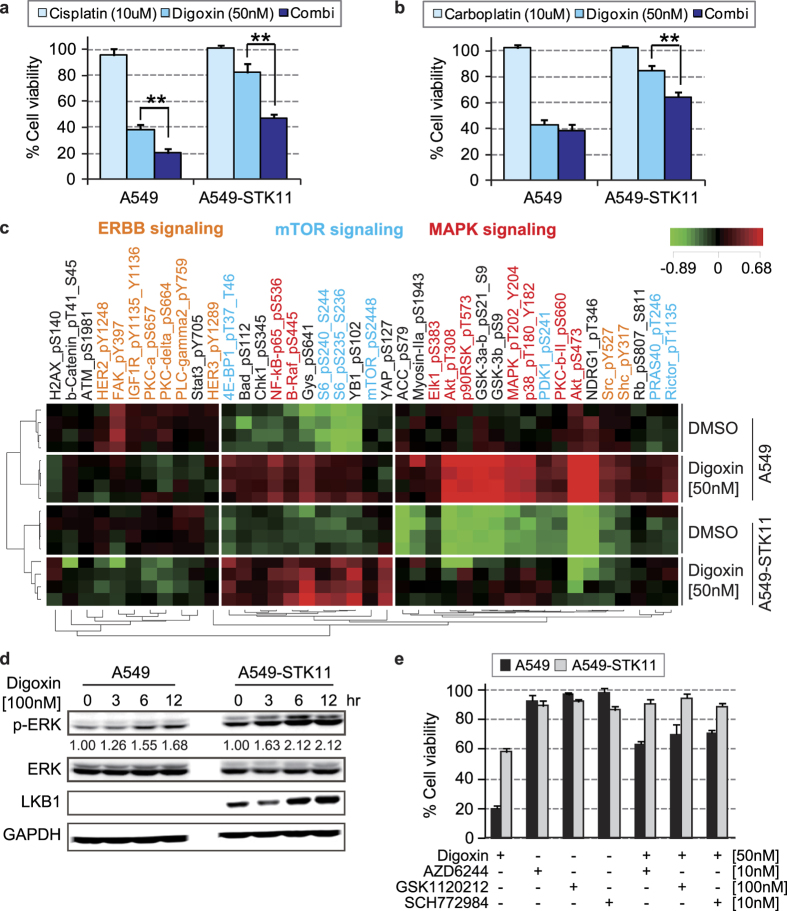
Investigation of the clinical potential of digoxin for the treatment of *STK11* mutant cancers. Synergistic effect of digoxin in combination (72 hours) with (**a)** cisplatin or (**b)** carboplatin on cell viability in *STK11* mutant and *STK11*-restored cell lines. (**c)** Proteomic analysis of the *STK11* mutant-specific effect of digoxin treatment. The median normalized log2 phosphorylation levels of 38 proteins (40 phosphorylation sites) based on the results of the RPPA experiment, were compared between A549 cells and their *STK11*-restored derivatives after digoxin treatment. Red color represents an increased phosphorylation level, and green represents a decreased phosphorylation level. The listed proteins satisfied the cutoff value of p < 0.01 (Student’s t-test) for the difference in phosphorylation between digoxin-treated and DMSO-treated cells (4 replicates each group). (**d)** CG induced ERK activation in A549 cells and *STK11*-restored A549 cells. ERK phosphorylation was measured after 3, 6, and 12 hours of treatment with 100 nM digoxin, and the quantified ERK phosphorylation level is shown as a numeric value. (**e)** Inhibiting ERK signaling reduces the effects of CGs. Cells were treated with inhibitors of the ERK pathway - AZD624, GSK212 or SCH772984 in the presence or absence of digoxin for 72 hours. The rate of cell viability was calculated using DMSO as a control for each inhibitor. Additionally, the rate of protein phosphorylation was calculated using GAPDH as a control. *p < 0.05 and **p < 0.01 (Student’s t-test) between the compared groups.

We subsequently investigated the combined effects of digoxin with cisplatin and carboplatin, which are FDA-approved chemotherapeutic agents for lung cancers. Particularly, *STK11* mutant cell lines were slightly more sensitive to cisplatin (Fig. 1d). Importantly, cisplatin, but not carboplatin, markedly increased the activity of digoxin in *STK11* mutant cells (Fig. 4a,b). The combination of vemurafenib (BRAF inhibitor) with digoxin has been tested in metastatic melanoma patients harboring *BRAF* mutations (Supplementary Table S3). In the present study, we found that *BRAF*-activating mutation was associated with resistance to CGs (Fig. 1b,c). Proteomic profiling showed increased expression and phosphorylation of proteins in the MAPK signaling pathway after digoxin treatment (Fig. 4c and Supplementary Fig. S5a). We confirmed that digoxin or ouabain treatment induced ERK activation in both the *STK11* mutant cell lines and their *STK11*-restored derivatives (Fig. 4d and Supplementary Fig. S5b–d). CGs have been proposed to induce cell apoptosis or autophagy by altering ERK signaling^23^,^24^. However, we found that the anti-cancer effect of digoxin treatment was decreased by inhibiting ERK signaling using AZD6244 (MEK1 inhibitor), GSK1120212 (MEK1/2 inhibitor) or SCH772984 (ERK inhibitor) (Fig. 4e, Supplementary Table S4 and Supplementary Fig. S5e). Thus, it appears that continued MEK-ERK signaling is important for digoxin-induced anti-cancer activity and that a combination strategy using digoxin is not promising as a MEK- or ERK-targeted cancer therapy.

### *STK11*-speicifc *in vivo* efficacy of digoxin

**Figure 5:**
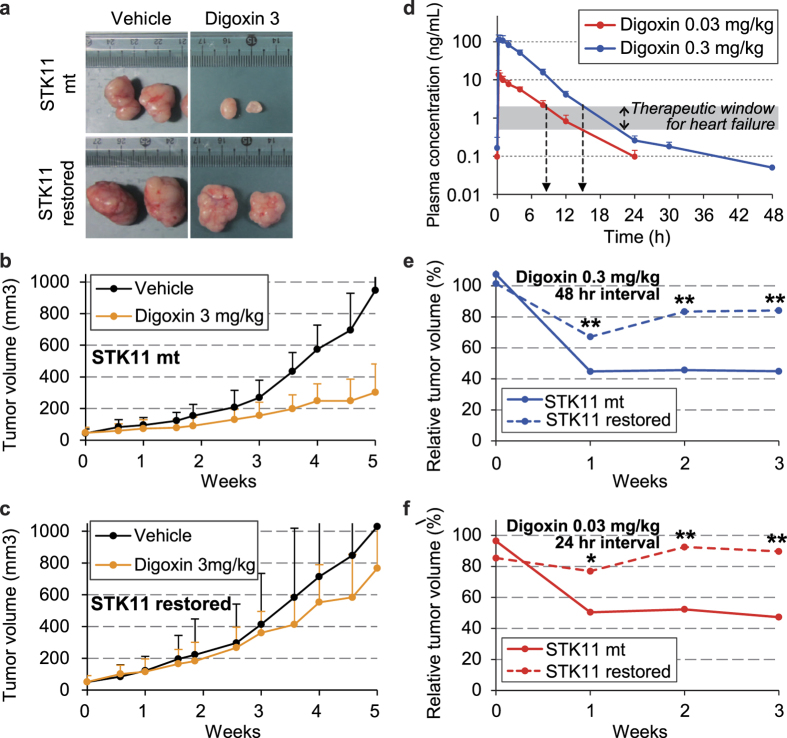
*In vivo* efficacy of digoxin on A549 (*STK11* mutant) and *STK11*-restored A549 cells. A549 and *STK11*-restored A549 cells were xenografted into nude mice. A total of 3 mg/kg (60 μg/ind.) digoxin was administrated via gavage every 48 hours for 5 weeks. The change in the tumor volume of the (**a**,**b)** A549 and (**a**,**c)** *STK11*-restored A549 xenografts was monitored. (**d)** The time course of the plasma concentration of digoxin was monitored after repeated oral administration of 0.3 mg/kg (6 ug/ind.) or 0.03 mg/kg (0.6 ug/ind.) digoxin at 48-hour and 24-hour intervals, respectively. The shaded area (0.5 ~ 2.0 ng/ml) represents the therapeutic blood concentration range of digoxin for clinical application to heart failure. Efficacy of a clinically relevant dose of digoxin. The tumor volume was compared between the A549 and *STK11*-restored A549 xenografts after (**e)** 0.3 mg/kg (6 μg/ind.) or (**f)** 0.03 mg/kg (0.6 μg/ind.) digoxin administration via gavage for 3 weeks every 48 hours or 24 hours, respectively. The relative tumor volume of each xenograft was calculated using vehicle administration as a control. *p < 0.05 and **p < 0.01 (Student’s t-test) between the compared groups.

To determine the *in vivo* efficacy of CGs on *STK11* mutant cancer, we utilized a mouse tumor xenograft model using A549 cells (*STK11* mutant) and their *STK11*-restored derivatives. Digoxin at achievable doses in mice (3 mg/kg)^25^ significantly time-dependently reduced tumor growth in mice injected with A549 cells (Fig. 5a,b) but not in mice injected with A549 cells transfected with wild type *STK11* (*STK11*-restored) (Fig. 5a,c). To investigate the clinical relevance of CG treatment in *STK11* mutant lung cancers, we further determined the dose required to maintain human-equivalent blood levels of digoxin in mice. This therapeutic range of CG concentrations is very narrow (0.5 ~ 2.0 ng/ml) for clinical applications^26^,^27^. To achieve this blood concentration in mice, the minimal dose of digoxin was 0.03 mg/kg and 0.3 mg/kg for administration at 24- and 48-hour intervals, respectively (Fig. 5d). At these doses and administration intervals, the plasma level of digoxin was maintained within (or below) the target range for ~70% of time period between administrations. In our second mouse xenograft experiment, 0.03 mg/kg digoxin at 24-hour intervals and 0.3 mg/kg digoxin at 48-hour intervals similarly resulted in a 30 ~ 40% difference in the tumor size between the A549 and A549 *STK11*-restored tumors (Fig. 5e,f). These results show that CGs exert their effects on tumors harboring an *STK11* mutation in a clinically relevant dose range.

### Mechanism for digoxin sensitivity of *STK11* mutant cancer cells

**Figure 6:**
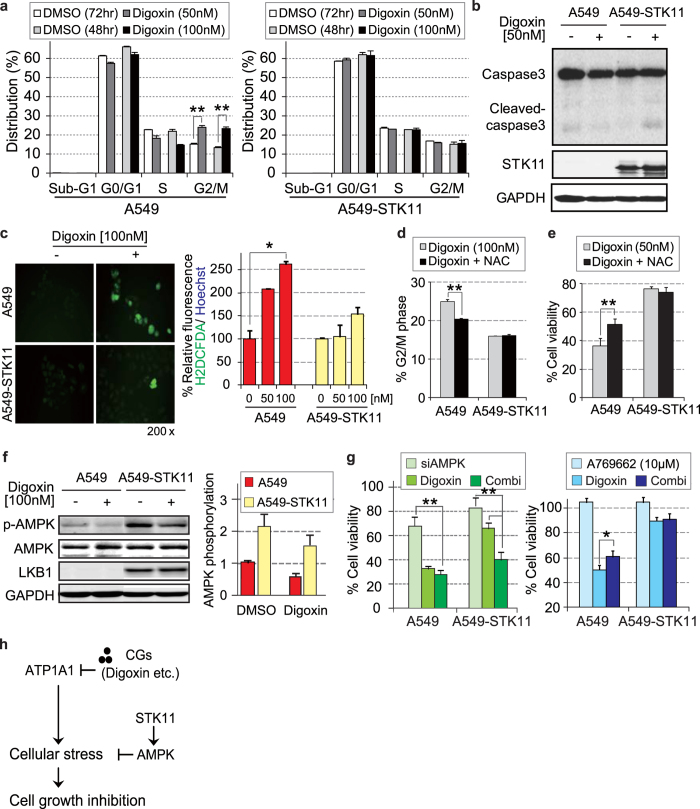
Mechanistic validation of the CG-mediated effect on *STK11* mutant cancer. (**a)** G2/M arrest was significantly induced in *STK11* mutant A549 cells by digoxin treatment. A549 and *STK11*-resotred A549 cells were treated with 50 nM and 100 nM digoxin for 72 and 48 hours, respectively. (**b)** Apoptosis was not induced by digoxin treatment. Caspase3 cleavage was detected after digoxin treatment (72 hours). Total proteins were immunoblotted with anti-caspase-3, anti-cleaved caspase-3 and anti-STK11 antibodies. (**c)** The ROS level was increased by digoxin treatment (48 hours) in *STK11* mutant A549 cells. The change in the level of ROS (green dots) was observed using a fluorescence microscope. (**d)** ROS inhibition attenuated digoxin-mediated G2/M arrest in *STK11* mutant A549 cells. Cells were incubated in digoxin (100 nM) for 48 hours combined with or without pretreatment of the ROS inhibitor NAC (1 mM) for 1 hour. (**e)** The influence of ROS inhibition on the efficacy of CGs. Cells were incubated in digoxin (50 nM) for 72 hours combined with or without pretreatment of the ROS inhibitor NAC (1 mM) for 1 hour. (**f)** AMPK signaling was sustained in *STK11*-restored A549 cells regardless of CG treatment. AMPK phosphorylation was measured after 6 hours of treatment with 100 nM digoxin, and the quantified AMPK phosphorylation level is shown on the right. (**g)** The influence of AMPK regulation on the efficacy of CGs. Cells were incubated in AMPK-targeted siRNA and A769662 (AMPK activator) with or without digoxin (50 nM) for 72 hours. (**h)** Proposed model for the effect of CGs on cellular signaling. STK11/AMPK signaling acts as a protective mechanism against CG-induced growth inhibition. The rate of cell viability was calculated using siNC and DMSO as a control for each siRNA and inhibitor, respectively. Additionally, the rate of protein phosphorylation was calculated using GAPDH as a control. *p < 0.05 and **p < 0.01 (Student’s t-test) between the compared groups.

We confirmed that digoxin treatment induced G2/M arrest more efficiently in *STK11* mutant A549 cell than in *STK11*-restored cells, but did not induce detectable apoptosis (Fig. 6a,b). Digoxin and other CGs block tumor growth by diverse mechanisms associated with altering ERK^23^,^24^ or HIF-1^25^,^28^ signaling. CGs induce cellular stress by increasing intracellular Ca^2+^ levels and blocking ATP hydrolysis^29^,^30^. CG-mediated generation of reactive oxygen species (ROS) is also responsible for the cytotoxicity of CG treatment^31^. We thus compared the changes in ROS levels between A549 and *STK11* restored A549 cell lines after digoxin treatment. Digoxin significantly induced the generation of ROS in *STK11* mutant cells but not in *STK11*-restored cells (Fig. 6c). Furthermore, the ROS inhibitor NAC significantly reduced digoxin-mediated G2/M arrest in *STK11* mutant A549 cells (Fig. 6d). Inhibition of ROS also attenuated the efficacy of digoxin in *STK11* mutant A549 cells (Fig. 6e), implying that the CG-induced increase in cellular stress was associated with the *STK11*-sensitive efficacy of CGs. Functional STK11-AMPK signaling is important for cancer cells to overcome stress responses induced by anti-cancer agents^1^,^32^,^33^. Particularly, AMPK activation inhibits the formation of ROS^34^. However, the phosphorylated AMPK levels were consistently lower in *STK11* mutant cells than in *STK11*-restored cells, regardless of digoxin treatment (Fig. 6f and Supplementary Fig. S6a). Applying siAMPK to *STK11* restored cells significantly increased their sensitivity to digoxin treatment (Fig. 6g and Supplementary Fig. S6b), and this observation implies that STK11-AMPK signaling is important for overcoming cellular stress induced by CGs. Furthermore, growth inhibition by digoxin treatment was attenuated in *STK11* mutant cells by activating AMPK using the direct AMPK agonist, A769662 (Fig. 6g and Supplementary Fig. S6c). The AMPK activation seems to provide a resistant mechanism against anti-cancer actions of CGs through induction of oxidative cell damage. Taken together, the results of the present study showed that *STK11* mutations sensitize cancer cells to CG treatment via dysregulation of cellular stress responses (Fig. 6h).

Despite the anticancer activities of CGs via ATP1A1 inhibition, CGs have not been used clinically due to a lack of biomarkers for the identification of patients who would be most likely to benefit from CG treatment (Supplementary Table S3). Our data mining and experimental validation results suggest that clinically relevant doses of CGs exert selective effects on *STK11* mutant cancer samples. *STK11* loss-of-function mutation is specifically observed in LUAD tissue samples and is mutually exclusive from alterations of known drug targets such as *ALK*, *BRAF*, and *EGFR*. These findings provide a novel selection approach to predict the utility of CGs for lung cancer patients with *STK11* mutant LUAD, who are most likely to benefit from CGs either alone or in concert with other therapies.

## Methods

### Acquisition of drug screening data

The therapeutic anticancer drug screening data for NCI-60 human tumor cell lines were obtained from the NCI/NIH Developmental Therapeutics Program (DTP) (www.dtp.nci.nih.gov). This dataset, released in July 2012, provides the GI_50_ values characterizing the sensitivity of NCI-60 DTP human tumor cell lines to 50,839 compounds^4^. The GI_50_ value is defined as the concentration of a compound required to inhibit cell growth by 50% at 48 hours after compound treatment compared with DMSO vehicle treatment. The DrugBank database has widely been used to facilitate drug discovery and *in silico* drug target discovery^35^.

### Acquisition and analysis of somatic mutation data

Analysis and visualization of genetic alterations in lung adenocarcinoma were performed using cBioPortal^36^,^37^. The details of data mining are provided in the Supplementary Methods.

### Cell line enrichment analysis

To quantify the association between the compound response (GI_50_) and a given mutation (e.g., *STK11* or *BRAF*), cell line enrichment analysis was applied as a statistical method^21^. The details of statistical methods are provided in the Supplementary Methods.

### siRNA transfection

The details of the siRNAs and experimental procedures used for transfection are provided in the Supplementary Methods.

### Real-time PCR

The details of the primers and experimental procedures used for real-time PCR are provided in the Supplementary Methods.

### Acquisition and analysis of shRNA screening data

The genome-scale shRNA library screening data for 216 cancer cell lines was obtained from the data portal of Project Achilles^13^. We used the gene-level shRNA scores in the Achilles version 2.4 dataset. The zygosity of *STK11* mutations in cancer cell lines was determined using somatic mutation data derived from the data portal of the Cancer Cell Line Encyclopedia (CCLE)^38^. The shRNA scores of ATP1A1 were compared between homozygous (NCI-H23, HCC44, PSN1, NCI-H2122, A549, NCI-H838 and JHOM1) and heterozygous (CAOV3, 22RV1, KALS1, A2058 and RKN) *STK11* mutant cell lines.

### Expression plasmid construction and transient transfection

The pLenti-LKB1-puro mammalian expression vector, a generous gift from Dr. Zhijun Luo (Boston University School of Medicine, Boston, MA, USA) was manipulated to construct the pBabe-puro-LKB1 expression vector. pLenti-LKB1-puro or pLenti-LKB1-mock was transiently transfected into HEK-293T cells to produce viral particles using a Thermo Scientific Trans-Lentiviral Packaging Kit (Dharmacon Inc.) in accordance with the guidelines of the institutional biosafety committee. Viral particles were infected into A549, NCI-H460, NCI-H23 and NCI-H1993 cells. The infected cells were selected using 2 μg/ml puromycin, and ectopic LKB1 expression was confirmed via western blot.

### Cell viability assay

The cells were seeded in a 96-well plate at a density of 2 × 10^3^ cells per well for 3 days, 1 × 10^3^ cells per well for 7 days or 200 cells per well for 14 days. After 24 hours in culture, the cells were treated with chemicals at the indicated concentrations. The cells were incubated for an additional 3, 7 or 14 days, and cell viability was measured using the CellTiter-Blue Cell Viability Assay (Promega, Madison, WI, USA). The culture medium was replaced every 3–4 days. The rate of cell viability was calculated using DMSO as a control for each chemical. And the percentage growth is calculated as: [(Ti-Tz)/(C-Tz)] × 100 for each treatment for which Ti > /=Tz or [(Ti-Tz)/Tz] × 100 for each treatment for which Ti < Tz, where Tz represents growth at time zero; C, control growth; Ti, test growth in the presence of chemical.

### Cell cycle analysis

A549 and *STK11*-restored A549 cells were treated with 100 nM digoxin for 48 hours. Cell distribution in the G1, S, and G2/M phases of the cell cycle was determined via fluorescence-activated cell sorting (FACS) analysis, and the results were analyzed using FlowJo software (FlowJo LLC, USA). Further details are provided in the Supplementary Methods.

### Invasion assay

To measure the effect of digoxin on cancer cell invasion, transwell cell culture chambers (Costar 3422, Cambridge, MA, USA) containing 12-μm pore size filters were used for cell invasion assays. Further details are provided in the Supplementary Methods.

### Soft agar colony formation assay

A549 and *STK11*-restored A549 cells were diluted to 5 × 10^3^ cells/well in 0.35% Noble agar (Sigma) solution in RPMI-1640 medium containing 10% FBS. Further details are provided in the Supplementary Methods.

### Serum-free 3D sphere formation assay

A549, NCI-H23, *STK11*-restored A549, H23 (2.5 × 10^3^ cells/well), NCI-H1993, and *STK11*-restored H1993 cells (5 × 10^3^ cells/well) was seeded in ultra-low attachment 96 well plates and cultured in CSC medium (20 ng/ml EGF, 20 ng/ml FGFB, 2% b-27, 1% penicillin/streptomycin and 10% FBS). After 5 days, the cells were stained with DAPI and cell spheres were counted (≧100 μm).

### BrdU incorporation assay

Details are provided in the Supplementary Methods.

### Measurement of ROS

Intracellular ROS production was determined by incubating the cells in 10 μM H2DCFDA and 1 μg/mL Hoechst 33342 (Sigma-Aldrich, USA) for 30 min at 37 °C in the dark. The cells were washed twice in PBS and analyzed using a Cytation3 cell imager (BioTek, USA) or observed using a fluorescence microscope (Olympus, Japan).

### RPPA

RPPA data for 223 pan- and 62 phosphorylation-specific antibodies (a total of 245 unique proteins) for DMSO and digoxin-treated A549 and *STK11*-restored cells were generated in the Functional Proteomics Core of the M.D. Anderson Cancer Center, University of Texas. Further details are provided in the Supplementary Methods.

### Western blot

The details of the antibodies and experimental procedures used for western blot are provided in the Supplementary Methods.

### Xenograft mouse model

Athymic nude mice were purchased from Oriental Bio Inc. (Guro-gu, Seoul, Korea) and were maintained under specific pathogen-free conditions in accordance with the guidelines approved by the Institutional Animal Care and Use Committee at the Catholic University of Korea (Approval numbers: 2014–005 and 2015–006). In the first *in vivo* experiment, 3 × 10^6^ A549 or *STK11*-restored A549 cells were subcutaneously injected into the right dorsal flank of athymic nude mice, which were housed until the tumor volume reached 50 mm^3^. The mice were divided into the following 2 treatment groups: vehicle alone (n = 10) or 60 μg digoxin (n = 10). Digoxin and vehicle were orally administered 3 times per week for 5 weeks. In the second experiment, 3 × 10^6^ A549 or *STK11*-restored A549 cells were subcutaneously injected into the right and left dorsal flanks of athymic nude mice, which were housed until the tumor volume reached 50 mm^3^. The mice were divided into the following 3 treatment groups: vehicle alone (n = 19), 0.6 μg digoxin (n = 20) or 6 μg (n = 19) digoxin. Vehicle and 6 μg digoxin were orally administered 3 times per week and 0.6 μg digoxin was orally administered 7 times per week for 3 weeks. Further details are provided in the Supplementary Methods.

### Pharmacokinetics of digoxin

Pharmacokinetic analysis of digoxin was conducted at the end of the *in vivo* pharmacodynamic experiment in accordance with the guidelines approved by the Institutional Animal Care and Use Committee at the Catholic University of Korea (Approval number: 2015–006). Male athymic nude mice were administered 0.6 or 6 μg/ind. digoxin. To measure the level of digoxin, blood samples (approximately 45 μL) were collected before administration and at 0.25, 0.5, 1, 2, 4, 8, 12, and 24 hours after administration for 0.6 μg/individuals or at 0.25, 0.5, 1, 2, 4, 8, 12, 24, 30, and 48 hours after administration for 6 μg/individuals. Plasma samples were treated and prepared for LC-MS/MS analysis. Further details are provided in the Supplementary Methods.

A detailed description of Methods is available in Supplementary Methods.

## Additional Information

**How to cite this article**: Kim, N. *et al*. Cardiac glycosides display selective efficacy for *STK11* mutant lung cancer. *Sci. Rep.* **6**, 29721; doi: 10.1038/srep29721 (2016).

## Supplementary Material

Supplementary Information

## References

[b1] Shackelford D. B. . LKB1 inactivation dictates therapeutic response of non-small cell lung cancer to the metabolism drug phenformin. Cancer Cell 23, 143–158 (2013). 23352126 10.1016/j.ccr.2012.12.008 PMC3579627

[b2] Ji H. . LKB1 modulates lung cancer differentiation and metastasis. Nature 448, 807–810 (2007). 17676035 10.1038/nature06030

[b3] He N. . Integrated analysis of transcriptomes of cancer cell lines and patient samples reveals STK11/LKB1-driven regulation of cAMP phosphodiesterase-4D. Mol Cancer Ther. 13, 2463–2473 (2014). 25122068 10.1158/1535-7163.MCT-14-0297

[b4] Reinhold W. C. . CellMiner: a web-based suite of genomic and pharmacologic tools to explore transcript and drug patterns in the NCI-60 cell line set. Cancer Res. 72, 3499–3511 (2012). 22802077 10.1158/0008-5472.CAN-12-1370 PMC3399763

[b5] Shoemaker R. H. The NCI60 human tumour cell line anticancer drug screen. Nat Rev Cancer 6, 813–823 (2006). 16990858 10.1038/nrc1951

[b6] Hollman A. Drugs for atrial fibrillation. Digoxin comes from Digitalis lanata. BMJ 312, 912 (1996). 8611904 10.1136/bmj.312.7035.912 PMC2350584

[b7] Yang P. . Oleandrin-mediated inhibition of human tumor cell proliferation: importance of Na,K-ATPase alpha subunits as drug targets. Mol Cancer Ther. 8, 2319–2328 (2009). 19671733 10.1158/1535-7163.MCT-08-1085

[b8] Fedorova O. V. & Bagrov A. Y. Inhibition of Na/K ATPase from rat aorta by two Na/K pump inhibitors, ouabain and marinobufagenin: evidence of interaction with different alpha-subunit isoforms. Am J Hypertens 10, 929–935 (1997). 9270089 10.1016/s0895-7061(97)00096-4

[b9] Sticherling C. . Effects of digoxin on acute, atrial fibrillation-induced changes in atrial refractoriness. Circulation 102, 2503–2508 (2000). 11076824 10.1161/01.cir.102.20.2503

[b10] Barry W. H., Hasin Y. & Smith T. W. Sodium pump inhibition, enhanced calcium influx via sodium-calcium exchange, and positive inotropic response in cultured heart cells. Circ Res. 56, 231–241 (1985). 2578900 10.1161/01.res.56.2.231

[b11] Mijatovic T. . Cardiotonic steroids on the road to anti-cancer therapy. Biochim Biophys Acta 1776, 32–57 (2007). 17706876 10.1016/j.bbcan.2007.06.002

[b12] Vaklavas C., Chatzizisis Y. S. & Tsimberidou A. M. Common cardiovascular medications in cancer therapeutics. Pharmacol Ther. 130, 177–190 (2011). 21277894 10.1016/j.pharmthera.2011.01.009

[b13] Cowley G. S. . Parallel genome-scale loss of function screens in 216 cancer cell lines for the identification of context-specific genetic dependencies. Sci Data 1, 140035 (2014). 25984343 10.1038/sdata.2014.35 PMC4432652

[b14] Ding L. . Somatic mutations affect key pathways in lung adenocarcinoma. Nature 455, 1069–1075 (2008). 18948947 10.1038/nature07423 PMC2694412

[b15] Kim N. . Differential regulation and synthetic lethality of exclusive RB1 and CDKN2A mutations in lung cancer. Int J Oncol. 48, 367–375 (2016). 26647789 10.3892/ijo.2015.3262 PMC6903902

[b16] He N. . Glucose starvation induces mutation and lineage-dependent adaptive responses in a large collection of cancer cell lines. Int J Oncol. 48, 67–72 (2016). 26573869 10.3892/ijo.2015.3242 PMC4734611

[b17] Bronte G. . Driver mutations and differential sensitivity to targeted therapies: a new approach to the treatment of lung adenocarcinoma. Cancer Treat Rev. 36 Suppl 3, S21–S29 (2010). 21129606 10.1016/S0305-7372(10)70016-5

[b18] de Castro Carpeno J. & Belda-Iniesta C. KRAS mutant NSCLC, a new opportunity for the synthetic lethality therapeutic approach. Transl Lung Cancer Res. 2, 142–151 (2013). 10.3978/j.issn.2218-6751.2013.02.07 PMC4369862 25806225

[b19] Kaelin W. G. Jr. The concept of synthetic lethality in the context of anticancer therapy. Nat Rev Cancer 5, 689–698 (2005). 16110319 10.1038/nrc1691

[b20] Jeong E. . MACE: mutation-oriented profiling of chemical response and gene expression in cancers. Bioinformatics 31, 1508–1514 (2015). 25536965 10.1093/bioinformatics/btu835

[b21] Kim N. . Systematic analysis of genotype-specific drug responses in cancer. Int J Cancer 131, 2456–2464 (2012). 22422301 10.1002/ijc.27529 PMC4012336

[b22] Katz A. . Selectivity of digitalis glycosides for isoforms of human Na, K-ATPase. J Biol Chem. 285, 19582–19592 (2010). 20388710 10.1074/jbc.M110.119248 PMC2885237

[b23] Wang Y. . Cardiac glycosides induce autophagy in human non-small cell lung cancer cells through regulation of dual signaling pathways. Int J Biochem Cell Biol. 44, 1813–1824 (2012). 22750415 10.1016/j.biocel.2012.06.028

[b24] Winnicka K., Bielawski K., Bielawska A. & Miltyk W. Apoptosis-mediated cytotoxicity of ouabain, digoxin and proscillaridin A in the estrogen independent MDA-MB-231 breast cancer cells. Arch Pharm Res. 30, 1216–1224 (2007). 18038900 10.1007/BF02980262

[b25] Zhang H. . Digoxin and other cardiac glycosides inhibit HIF-1alpha synthesis and block tumor growth. Proc Natl Acad Sci. USA 105, 19579–19586 (2008). 19020076 10.1073/pnas.0809763105 PMC2604945

[b26] Miura T. . Effect of aging on the incidence of digoxin toxicity. Ann Pharmacother. 34, 427–432 (2000). 10772425 10.1345/aph.19103

[b27] Adams K. F. Jr. . Relationship of serum digoxin concentration to mortality and morbidity in women in the digitalis investigation group trial: a retrospective analysis. J Am Coll Cardiol. 46, 497–504 (2005). 16053964 10.1016/j.jacc.2005.02.091

[b28] Samanta D., Gilkes D. M., Chaturvedi P., Xiang L. & Semenza G. L. Hypoxia-inducible factors are required for chemotherapy resistance of breast cancer stem cells. Proc Natl Acad Sci. USA 111, E5429–E5438 (2014). 25453096 10.1073/pnas.1421438111 PMC4273385

[b29] Kepp O. . Anticancer activity of cardiac glycosides: At the frontier between cell-autonomous and immunological effects. Oncoimmunology 1, 1640–1642 (2012). 23264921 10.4161/onci.21684 PMC3525630

[b30] Soltoff S. P. & Hedden L. Regulation of ERK1/2 by ouabain and Na-K-ATPase-dependent energy utilization and AMPK activation in parotid acinar cells. Am J Physiol Cell Physiol. 295, C590–C599 (2008). 18632735 10.1152/ajpcell.00140.2008 PMC2544437

[b31] Prassas I. & Diamandis E. P. Novel therapeutic applications of cardiac glycosides. Nat Rev Drug Discov. 7, 926–935 (2008). 18948999 10.1038/nrd2682

[b32] Alexander A. & Walker C. L. The role of LKB1 and AMPK in cellular responses to stress and damage. FEBS Lett. 585, 952–957 (2011). 21396365 10.1016/j.febslet.2011.03.010

[b33] Carretero J. . Dysfunctional AMPK activity, signalling through mTOR and survival in response to energetic stress in LKB1-deficient lung cancer. Oncogene. 26, 1616–1625 (2007). 16953221 10.1038/sj.onc.1209951

[b34] Shirwany N. A. & Zou M. H. AMPK in cardiovascular health and disease. Acta Pharmacol Sin. 31, 1075–1084 (2010). 20711221 10.1038/aps.2010.139 PMC3078651

[b35] Law V. . DrugBank 4.0: shedding new light on drug metabolism. Nucleic Acids Res. 42, D1091–D1097 (2014). 24203711 10.1093/nar/gkt1068 PMC3965102

[b36] Gao J. . Integrative analysis of complex cancer genomics and clinical profiles using the cBioPortal. Sci. Signal 6, pl1 (2013). 10.1126/scisignal.2004088 PMC4160307 23550210

[b37] Cerami E. . The cBio cancer genomics portal: an open platform for exploring multidimensional cancer genomics data. Cancer Discov. 2, 401–404 (2012). 22588877 10.1158/2159-8290.CD-12-0095 PMC3956037

[b38] Barretina J. . The Cancer Cell Line Encyclopedia enables predictive modelling of anticancer drug sensitivity. Nature 483, 603–607 (2012). 22460905 10.1038/nature11003 PMC3320027

