# Supplementary material for: Cardiac glycosides display selective efficacy for STK11 mutant lung cancer
Source: Sci Rep. 2016 Jul 19;6:29721. doi: 10.1038/srep29721 (PMC4949473; doi:10.1038/srep29721)
Supplement: Supplementary Information [file srep29721-s1.doc]

**Supplementary information**

**Cardiac glycosides display selective efficacy for *STK11* mutant lung cancer**

Nayoung Kim^1,2,†^, Hwa Young Yim^1,†^, Ningning He^2^, Cheol-Jung Lee^3^, Ju Hyun Kim^3^, Jin-Sung Choi^3^, Hye Suk Lee^3^, Somin Kim^1^**,** Euna Jeong^1^, Mee Song^1^, Sang-Min Jeon^4^, Woo-Young Kim^5^, Gordon B Mills^6^, Yong-Yeon Cho^3^, Sukjoon Yoon^1,2,*^

**Contents**

Supplementary Methods

Supplementary Figures S1 – S6

Supplementary Tables S1 – S4

**Supplementary Methods**

**Reagents and antibodies**

The chemical reagents NaCl, Tris, glycine, sodium dodecyl sulfate (SDS), ammonium persulfate (APS), N,N,N,N-tetramethylethylenediamine (TEMED), and 30% acrylamide were purchased from Bio-Rad (Richmond, CA, USA). The therapeutic agents digoxin, digitoxin, ouabain and NAC were purchased from Sigma-Aldrich (St. Louis, MO, USA). AZD6244, GSK1120212, SCH772984, cisplatin, and carboplatin were purchased from AbMole BioScience (Hong Kong, China). A769662 was purchased from Selleck Chemicals LLC (Houston, TX, USA). [Dimethyl sulfoxide (DMSO) was purchased from](http://europepmc.org/abstract/med/3510103) Calbiochem, and puromycin was purchased from Sigma-Aldrich. Antibodies against phosphorylated-AMPK, phosphorylated-ERK, AMPK, ERK, LKB1, caspase-3, cleaved caspase-3 and GAPDH were purchased from Cell Signaling Technology (Beverly, MA, USA). RPMI-1640 cell culture medium and fetal bovine serum (FBS) were purchased from HyClone (USA). Cell culture supplements were purchased from Life Science Technology (Rockville, MD, USA) and Corning (Corning, NY, USA).

**Acquisition and analysis of somatic mutation data**

The somatic mutation and clinical data were obtained from the data portal of TCGA. This library included 538 LUAD and 178 lung squamous cell carcinoma (LUSC) patient tissue samples. The tumor tissue samples were categorized into four cancer stages (Stage I, II, III, and IV) based on the ‘pathologic_stage’ information in the clinical dataset. The frequency of *STK11* mutations was calculated for each cancer stage.

**Cell line enrichment analysis**

The prioritization of cell lines harboring a particular mutation according to the GI_50_ was analyzed using a receiver operator characteristic (ROC) curve. The area under the ROC curve (AUC) was used as a measure of the accuracy of the association of a mutation with the given compound response. The significance (p-value) of the AUC was assigned using permutation tests via 1,000 repeated randomizations of the ranked mutation list.

**Cell culture**

NCI-60 lung cancer cell lines (NCI-H460, A549, NCI-H322M, NCI-H23 and NCI-H226) were obtained from the National Cancer Institute (NCI DTP). The NCI-H1993, NCI-H1395, NCI-H82, and NCI-H524 cell lines were obtained from American Type Culture Collection (ATCC). All cells were cultured in RPMI-1640 medium supplemented with 10% FBS according to institutional laboratory safety guidelines. The cells were maintained at 37°C in a 5% CO_2_ incubator and were subcultured at 80-90% confluence. The media were changed every other day.

**Invasion assay**

To measure the effect of digoxin on cancer cell invasion, transwell cell culture chambers (Costar 3422, Cambridge, MA, USA) containing 12-µm pore size filters were used for cell invasion assays. A total of 5 x 10^4^ cells were seeded on the upper compartment of the transwell cell culture chamber and were cultured for 24 hours. Then, the cells that had migrated to the lower compartment of the transwell cell culture chamber were counted in 10 random fields under a phase-contrast microscope (100X).

**BrdU incorporation assay**

A total of 2.5 x 10^3^ cells were seeded on 96-well plates and cultured for 24 hours to allow for cell attachment. The cells were pretreated with digoxin for 24 hours and then co-treated with 100 µM BrdU and digoxin for 2 hours. The incorporation of BrdU into genomic DNA was measured using a colorimetric BrdU detection kit and an ELISA plate reader (Roche, Basel, Switzerland).

**Soft agar colony formation assay**

A549 and *STK11*-restored A549 cells were diluted to 5 × 10^3^ cells/well in 0.35% Noble agar (Sigma) solution in RPMI-1640 medium containing 10% FBS. The cell suspension was added to each well of a 6-well plate coated with 0.5% Noble agar in RPMI-1640 medium containing 10% FBS at 37°C. Fresh RPMI-1640 medium containing 10% FBS was added to the top of the soft agar, and the cells were then cultured at 37°C for 14 days. The culture medium was replaced twice per week. The colonies were stained with a 0.05% crystal violet solution (dissolved in PBS; Sigma), and the positively stained colonies were counted. Each experiment was performed in triplicate.

**Cell cycle analysis**

The cells were harvested using Cell Dissociation Solution (Non-enzymatic, Sigma, USA) and washed twice with cold PBS. At total of 2 x 10⁶ cells were resuspended in 1 ml of cold PBS, and 9 ml of 70% EtOH was then slowly added dropwise to the cell suspension with gentle vortexing. This mixture was stored at 4°C for 24 hours, and the cells were washed with cold PBS. For staining with propidium iodide (PI), cells were resuspended in 300~500 μl PI/Triton X-100 staining solution (0.1% Triton X-100, 0.1% EDTA, 50 μg/ml RNase A, and 50 μg/ml PI (Sigma, USA) in PBS) and incubated at room temperature for 30 min in the dark. Fluorescence-activated cell sorting (FACS) analysis was performed using the FACSCanto II system (Becton Dickinson, USA), and cell distribution in the G1, S, and G2/M phases of the cell cycle was analyzed using FlowJo software (FlowJo LLC, USA).

**RPPA**

All cells were cultured in RPMI-1640 medium supplemented with 10% FBS. The cells were maintained at 37°C in a 5% CO2 incubator. Before protein harvesting, the cells were treated with DMSO and digoxin (50 nM) for 6 hours. RPPA assays were performed according to institutional protocols.

**Western blot**

Samples containing equal amounts of protein were resolved via SDS-polyacrylamide gel electrophoresis and transferred to PVDF membranes. The membranes were blocked with TBST [20 mM Tris-HCl, pH 7.6] containing 5% bovine serum albumin (BSA) and then hybridized to specific primary antibodies as indicated overnight. The membranes were washed and hybridized to HRP-conjugated secondary antibodies for 2 hours at room temperature. Then, specific bands were visualized using an enhanced chemiluminescence (ECL) detection system (Thermo, Logan, UT, USA) and an LA3000 luminescence image analyzer (Fujifilm, Tokyo, Japan).

**siRNA transfection**

A total of 3 x 10^3^ cells per well were seeded on a 96-well plate for viability assays, or 1 x 10^5^ cells per well were seeded on a 6-well plate for RNA extraction. After culturing overnight, the cells were transfected with siRNA targeting STK11 (L-005035-00, Dharmacon Inc.) or ATP1A1 (1009769, Bioneer Co., Ltd.) or with non-targeted siRNA (D-001810-01, Dharmacon Inc.) using an siRNA transfection kit purchased from Santa Cruz Biotechnology (Santa Cruz, CA, USA) in accordance with the manufacturer's protocol.

**Real-time PCR**

Total RNA was extracted from siRNA-transfected cells using Trizol (Invitrogen, Carlsbad, CA, USA), and cDNA synthesis and PCR amplification were performed using the Superscript One-Step RT-PCR Platinum Taq kit (Invitrogen, Carlsbad, CA, USA). The gene expression levels of STK11 and ATP1A1 were quantified via real-time PCR using specific primer sets targeting STK11 (Hs00176092_m1), ATP1A1 (Hs00167556_m1), and ACTIN (Hs99999903­_m1) and an Applied Biosystems 7500 system according to the manufacturer’s protocols. The CT values for STK11 and ATP1A1 RNA expression were normalized to the CT values for the RNA expression of the internal control ACTIN to evaluate equal amounts of RNA.

**Xenograft mouse model**

The tumor volume was calculated from measurements of 2 diameters of the individual tumor base using the following formula: tumor volume (mm^3^) = (length X width X height X 0.52). The mice were monitored until the tumors reached a total volume of 1 cm^3^, at which time the mice were euthanized and the tumors were extracted. Outliers in each group were removed from the statistical analysis.

**Pharmacokinetics of digoxin**

Plasma samples were treated and prepared for LC-MS/MS analysis. A 10-µL aliquot was injected into the Agilent 1290 UPLC system (Agilent Technologies, Wilmington, DE, USA), which fed the samples into a 6495 triple-quadrupole mass spectrometer (Agilent Technologies) equipped with an electrospray ionization source (ESI). Chromatographic separation was performed using a Halo C_18_ column (2.1 x 50 mm, 2.7 µm; Advanced Materials Technology, USA) at a flow rate was 0.3 mL/min and at autosampler and column temperatures of 4°C and 30°C, respectively. The ESI source settings for ionization of digoxin and ezetimibe (IS) in negative mode were as follows: drying gas temperature, 230°C; drying gas flow, 16 L/min; nebulizer gas pressure, 10 psi; sheath gas temperature, 300°C; sheath gas flow, 11 L/min; capillary voltage, 4000 V; and nozzle voltage, 1000 V. Fragmentation of digoxin and IS was performed using nitrogen as a collision gas. The collision energy (CE) and selective reaction monitoring (SRM) transitions for the quantification were as follows: 825.3 > 779.2 at CE 22 for digoxin and 408.0 > 271.0 at CE 12 for ezetimibe (IS). MassHunter software (Agilent Technologies) was used for LC-MS/MS system quality control and data processing.

**Statistical analysis**

For all experimental results, the significance of the differences between two groups was calculated using Student’s t-test. The data are expressed as the average values and standard error.

**Supplementary Figures**

**Supplementary Figure S1. Frequency of *STK11* mutations in lung cancer.**

The frequency of *STK11* mutations across cancer stages (I, II, III, and IV) among **a,** 538 lung adenocarcinoma and **b,** 178 squamous cell carcinoma tissue samples. The number on the plot indicates the number of samples. The frequency was calculated as the ratio of *STK11* mutant-harboring samples to all samples at each stage.

**Supplementary Figure S2. Ectopic LKB1 expression in *STK11*-restored cell lines.**

Western blotting was used to measure wild type LKB1 expression after transfection of the pLenti-LKB1-puro mammalian expression vector into *STK11* mutant (A549, NCI-H460, NCI-H23 and NCI-H1993) cell lines.

**Supplementary Figure S3. *STK11* mutant-specific response to cardiac glycosides.**

Changes in cell viability caused by digitoxin or ouabain treatment in the **a,** A549 and **b,** NCI-H460 cell lines between the *STK11* mutant and *STK11*-restored cells. The cells were treated with 25, 50, or 100 nM of a CG to inhibit ATP1A1 for 3 days. Long-term effect of digitoxin or ouabain on cell viability in the **c,** A549 and **d,** NCI-H460 cell lines between the *STK11* mutant and *STK11*-restored cells. The differential viability of the *STK11*mt cell line is presented as the difference in viability of the *STK11* mutant and cell lines relative to the *STK11*-restored cell line. The cells were treated with 50 nM digitoxin or 25 nM ouabain for 3, 7 or 14 days. Changes in cell division after CG treatment in the **e,** A549 and **f,** NCI-H460 cell lines between the *STK11* mutant and *STK11*-restored cells. The cells were treated with 100 nM digoxin or 50 nM digitoxin or ouabain for 24 hours. The percentages of cells incorporating BrdU and of viable cells were calculated using DMSO as a control. *p<0.05 and **p<0.01 (Student's t-test) between the compared groups.

**Supplementary Figure S4. Knockdown efficiency of siRNA treatment.**

qPCR was used to measure the decrease in gene expression after siRNA treatment. **a,** Treatment of the *STK11* mutant (A549 and H460), *STK11*-restored (A549-STK11 and H460-STK11), and *STK11* wild type (H322M and H226) cell lines with siRNA targeting *STK11*. **b,** Treatment of the same 8 cell lines with siRNA targeting *ATP1A1*.

**Supplementary Figure S5. CG-mediated changes in ERK signaling.**

**a,** Digoxin-dependent changes in the total protein levels in A549 and *STK11*-restored A549 cells. The median normalized log2 expression levels of 104 proteins based on the RPPA experiment was compared following digoxin treatment between A549 and *STK11*-restored A549 cells. Red color represents increased expression, and green represents decreased expression. The listed proteins satisfied the cutoff value of p<0.01 (Student's t-test) for the difference between digoxin and DMSO in 4 replicates for each cell line. **b,** Digoxin induced ERK signaling in both H460 and *STK11*-restored H460 cells. ERK phosphorylation was measured after 3, 6, and 12 hours of treatment with 100 nM digoxin. Ouabain induced ERK signaling in **c,** A549 and **d,** H460 cells and in the corresponding *STK11*-restored cells. ERK phosphorylation was measured after 3, 6, and 12 hours of treatment with 50 nM ouabain. The quantified ERK phosphorylation level is shown as a numeric value. **e,** Cells were treated with three inhibitors of the ERK pathway - AZD6244 (10 nM), GSK212 (100 nM) or SCH772984 (10 nM) - in the presence or absence of digoxin (50 nM) for 72 hours. The rate of cell viability was calculated using DMSO as a control for each inhibitor. Additionally, the rate of protein phosphorylation was calculated using GAPDH as a loading control. *p<0.05 and **p<0.01 (Student's t-test) between the compared groups.

**Supplementary Figure S6**. **Association of AMPK with CG-induced growth inhibition.**

**a,** AMPK phosphorylation was measured after 6 hours of treatment with 100 nM digoxin, and the quantified AMPK phosphorylation level is shown on the right. **b,** AMPK knockdown using siRNA enhanced the efficacy of CGs in *STK11*-restored H460 cells. **c,** Activating AMPK using A769662 (AMPK activator) attenuated the efficacy of CGs in *STK11* mutant H460 cells. Cells were incubated in AMPK-targeted siRNA/A769662 (10 µM) with or without digoxin (50 nM) for 72 hours. The rate of cell viability was calculated using siNC and DMSO as controls for each siRNA and inhibitor, respectively. Additionally, the rate of protein phosphorylation was calculated using GAPDH as a loading control. *p<0.05 and **p<0.01 (Student's t-test) between the compared groups.

**Supplementary Tables**

**Supplementary Table S1. Mutation-specific targeted therapeutic agents for lung cancer tested in clinical trials.**

| **Mutant gene** | **Mutation type** | **Targeted therapeutic agents tested in clinical trials** |
| --- | --- | --- |
| ALK | ALK fusions | First-generation ALK tyrosine kinase inhibitor (TKI) (Crizotinib)  Second-generation ALK TKIs (AP26113, CH5424802, Ceritinib)  HSP-90 inhibitors (Retaspimycin, Ganetespib) |
| BRAF | c.1415A>G (Y472C) | ABL/SRC/KIT inhibitor (Dasatinib) |
|  | c.1799T>A (V600E) | BRAF inhibitors (Vemurafenib, Dabrafenib) |
| EGFR | c.2156G>C (G719A)  c.2155G>T (G719C)  c.2155G>A (G719S) | EGFR TKIs (Erlotinib, Gefitinib) |
|  | Exon 19 deletion  c.2369C>T (T790M)  c.2573T>G (L858R) | First-generation EGFR TKIs (Erlotinib, Gefitinib)  Second-generation EGFR TKIs (Afatinib, Dacomitinib, Neratinib)  Third-generation (mutant-selective) EGFR TKIs  (CO-1686, AZD9291, AP26113, HM61713) |
| MET | MET amplification | MET/ALK TKI (Crizotinib) |
| RET | RET fusions | RET TKI (Cabozantinib) |
| ROS1 | ROS1 fusions | MET/ALK/ROS1 TKI (Crizotinib) |

**Supplementary Table S2. Details of mutational status of the tested STK11 mutant cell lines.**

| **Cell line** | **Lineage** | **Variant classification** | **cDNA change** | **Protein change** | **Mutation zygosity** | **KRAS mutation** |
| --- | --- | --- | --- | --- | --- | --- |
| A549 | LUNG|NSCLC | Nonsense_Mutation | c.109C>T | p.Q37* | hom | p.G12S |
| NCIH23 | LUNG|NSCLC | Nonsense_Mutation | c.996G>A | p.W332* | hom | p.G12C |
| NCIH460 | LUNG|NSCLC | Nonsense_Mutation | c.109C>T | p.Q37* | hom | p.Q61H |
| NCIH1993 | LUNG|NSCLC | Nonsense_Mutation | c.595G>T | p.E199* | hom | wt |
| NCIH1395 | LUNG|NSCLC | Frame_Shift_Del | c.165delG | p.L55fs | hom | wt |

**Supplementary Table S3. Current state of clinical trials for digoxin.**

| **Title** | **Investigator** | **Phase** | **Status** |
| --- | --- | --- | --- |
| Effect of Regorafenib on Digoxin and Rosuvastatin in Patients With Advanced Solid Malignant Tumors | Bayer | Phase 1 | active,  not recruiting |
| Study To Examine The Effects Of Lapatinib On The Pharmacokinetics Of Digoxin In Subjects w/ ErbB2 Positive Breast Cancer | GlaxoSmithKline | Phase 1 | completed |
| Capecitabine With Digoxin for Metastatic Breast Cancer | Western Regional Medical Center | Phase 2 | recruiting |
| DIG-HIF1 Pharmacodynamic Trial in Newly Diagnosed Operable Breast Cancer | Sidney Kimmel Comprehensive Cancer Center | Phase 2 | recruiting |
| Second Line Erlotinib (Tarceva) Plus Digoxin in Non-Small Cell Lung Cancer | James Graham Brown Cancer Center | Phase 2 | terminated |
| Drug-drug Interaction Study of Tivantinib (ARQ 197) With Omeprazole, S-warfarin, Caffeine, Midazolam, and Digoxin in Cancer Subjects | Daiichi Sankyo Inc. | Phase 1 | completed |
| Digoxin for Recurrent Prostate Cancer | Thomas Jefferson University | Phase 2 | completed |
| A Pharmacokinetics Study to Investigate the Effect of Vemurafenib on Digoxin in Patients With BRAFV600 Mutation-Positive Metastatic Melanoma | Hoffmann-La Roche | Phase 1 | completed |
| A Phase 1B Clinical Trial of Trametinib Plus Digoxin in Patients With Unresectable or Metastatic BRAF Wild-type Melanoma | University of Texas Southwestern Medical Center | Phase 1 | recruiting |
| Phase II Multicentric Study of Digoxin Per os in Classic or Endemic Kaposi' s Sarcoma | Assistance Publique - Hôpitaux de Paris | Phase 2 | recruiting |

**Supplementary Table S4. Inhibitors of the RAF/ERK signaling pathway.**

| **Mechanism of action** | **Name** | **IC50** |
| --- | --- | --- |
| Pan-MEK inhibitor | GSK1120212 | 0.92nM /1.8nM (MEK1/2) |
| Selective MEK inhibitor | AZD6244 | 10nM (MEK1) |
| ERK inhibitor | SCH772984 | 4nM /1nM (ERK1/2) |
